# Global crotonylome reveals hypoxia-mediated lamin A crotonylation regulated by HDAC6 in liver cancer

**DOI:** 10.1038/s41419-022-05165-1

**Published:** 2022-08-17

**Authors:** Dan Zhang, Jing Tang, Yunhong Xu, Xiaoju Huang, Yilin Wang, Xin Jin, Gang Wu, Pian Liu

**Affiliations:** 1 Cancer center, Union Hospital, Tongji Medical College, Huazhong University of Science and Technology, Wuhan, 430022 China; 2 Institute of Radiation Oncology, Union Hospital, Tongji Medical College, Huazhong University of Science and Technology, Wuhan, 430022 China; 3 Department of Urology, The Second Xiangya Hospital, Central South University, Changsha, Hunan 410011 China; 4 Uro-Oncology Institute of Central South University, Changsha, Hunan 410011 China

**Keywords:** Liver cancer, Oncogenes

## Abstract

Lysine crotonylation is a recently discovered post-translation modification involved in transcription regulation, cell signal transduction, and other processes. Scientists have identified several crotonylases and decrotonylases of histones, including P300/CBP, HDACs, and SIRTs. However, the regulation of non-histone protein crotonylation remains unclear. In the current study, we verified that crotonylation was upregulated in hypoxia and promoted liver cancer cell growth. We performed TMT-labeled quantitative lysine crotonylome analysis in 12 pairs of hepatocellular carcinoma and adjacent liver tissue and identified 3,793 lysine crotonylation sites in 1,428 proteins. We showed that crotonylation of lamin A at the site of K265/270 maintains its subcellular position, promotes liver cancer cell proliferation, and prevents cellular senescence. Our data indicate that HDAC6 is the decrotonylase of lamin A and downregulated in response to hypoxia, resulting in lamin A K265/270cr. Taken together, our study reveals the lamin A crotonylation in liver cancer progression and fills the research gap in non-histone protein crotonylation function.

## Introduction

Protein posttranslational modifications (PTMs), including phosphorylation, acetylation, ubiquitinoylation, methylation, crotonylation, and others, play a vital role in cellular signal transduction, which diversifies the cellular function and enables cells to adapt to various extracellular stimulation. Dysregulated PTMs can cause cell dysfunction and eventually lead to pathological processes such as carcinogenesis and metastasis. Classical acetylation is closely related to gene transcription regulation, cell cycle regulation, protein folding and degradation, RNA processing, and so on [1]. Recent studies have verified the existence of several new short-chain lysine acylations with the application of LC-MS/MS proteomic analysis, such as β-hydroxybutyrylation [2], butyrylation [3], crotonylation [4], succinylation [5], and glutarylation [6]. However, the cellular functions of these PTMs are not well understood.

Crotonylation was originally identified as a PTM on histone lysine residues. It was reported to be enriched on sex chromosomes and involved in the male germ cell differentiation process [4]. A series of subsequent studies revealed that crotonylation plays a further role in DNA damage and repair [7], self-renewal and differentiation of stem cells [8], HIV latency [9], cardiac homeostasis [10], carcinogenesis, and other processes by influencing protein structure and modulating protein stability, localization, and protein interactions. Lysine crotonylation (Kcr) may happen either enzymatically or nonenzymatically. Increasing the concentration of crotonyl-coA, the precursor of Kcr, can raise the average level of Kcr [11]. Histone acetyltransferases p300/CBP, MOF, and pCAF have also been discovered to be crotonyltransferasely active [12], and histone deacetylases including HDAC1/2/3 and SIRT1/2/3 show crotonylase activity [13]. Yet, the environmental stimulus leading to protein crotonylation has not been elucidated.

Due to the intensive proliferation and high oxygen demand in tumor tissue, hypoxia becomes a common feature of most solid tumors. Hypoxia changes the tumor behavioral patterns by altering metabolism pathways, promoting tumor angiogenesis, desensitizing chemotherapy or radiotherapy, and creating an immunosuppressive microenvironment. Hypoxia may seem toxic to cancer cells, but it appears to induce cancer cell malignant transformation by altering protein degradation, protein–protein interactions, RNA processing, and so on. However, how hypoxia affects liver cancer cells remains to be further investigated.

In the current study, we identified hypoxia as a potential inducer to promote crotonylation in liver cancer because the crotonylation level is tightly regulated by hypoxia and positively related to the expression of HIF1a. We further used quantitative proteomics technology to obtain a global analysis of the crotonylome in liver cancer and adjacent liver tissue. We identified 2,229 Kcr sites in 1,428 crotonylated proteins in these tissues. We took an intersection of the IP-MS and quantitative proteomics data and found that lamin A is crotonylated at K265/270. In particular, we characterized the functional significance of K265/270 R of lamin A regulated by HDAC6 and demonstrated a vital role of it in bypassing senescence by downregulating the expression of p21 and p16.

## Materials and methods

### Antibodies and Western blot analysis

The antibodies used in this research were as follows: pan-Kcr (PTM-501, PTM-502), anti-β-Tubulin (proteintech, 66240-1-Ig), anti-HIF1α (proteintech, 20960-1-AP), anti-Flag (proteintech, 20543-1-AP; ABclonal, AE005), anti-Lamin A/C (proteintech, 10298-1-AP), anti-Ki-67 (proteintech, 27309-1-AP), anti-HDAC6 (proteintech, 12834-1-AP), anti-6*HIS tag (66005-1-Ig), anti-p21 (proteintech, 60214-1-Ig), anti-p16 (Abcam, ab108349), HRP-conjugated Goat Anti-Mouse IgG (H + L) (SA00001-1), HRP-conjugated Goat Anti-Rabbit IgG (H + L) (SA00001-2). Tissues and cells were placed on ice and lysed by RIPA Lysis Buffer (Beyotime, P0013) with 1% Protease Inhibitor Cocktail (GLPBIO, GK10019). The lysis was rotated at 4 °C for 30 mins and boiled with SDS loading buffer for 10 mins at 95 °C. Western blots were performed following standard protocol.

### Cell lines and reagents

PLC/PRF/5, HepG2, Huh7, and sk-HEP1 cells were obtained from Boster Biological Technology and authenticated by the short tandem repeat test. A complete culture medium was prepared as follows: 10% FBS (BCI), 100 U/mL penicillin, and 100 μg/mL streptomycin were added to DMEM medium (Gbico). All cells used in this study were cultured in a humidified incubator with 5% CO_2_. Cells were cultured in 1% O_2_, 5% CO_2_, and 94% N_2_ for 12 h to induce hypoxia. Glucose-free DMEM was used for glucose starvation.

Crotonic acid was purchased from Tokyo Chemical Industry (CAS RN:107-93-7). Sodium crotonate (NaCr) was prepared as follows: 0.86 g crotonate acid was added to 9 mL ddH_2_O, followed by titration with sodium hydroxide to pH 7.35 in a final volume of 10 mL. In total, 10 mM of sodium crotonate was used to treat cultured cells and 12 mmol/kg body wt to treat mice. HDAC inhibitors or histone acetyltransferase inhibitors were obtained from MedChemExpress company.

### Molecular cloning

The human LMNA gene was cloned into a pcDNA3.1-3xFlag tag vector. Pre-lamin A and the recombinant of lamin A containing 1-385,1-308, and 1-241 amino acids were subcloned into a pcDNA3.1-3FLAG vector. The wild-type lamin A and mutants were cloned into a pmCherry-C1 vector, and a K265/270 R mutant was subcloned into a pcDNA3.1.3xFlag tag vector. The gRNAs targeting LMNA exon2 were cloned into the lentiCRISPR-v2 vector. Human HDAC6 gene was cloned into a pcDNA3.1-6xHIS vector. The DNA fragments were purified by EZ-10 Column DNA Purification Kit (Sangon Biotech, Shanghai), and the plasmids were extracted by SanPrep Endotoxin-Free Plasmid Mini Kit (Sangon Biotech, Shanghai). The primers used in molecular cloning and plasmid construction are listed in Table S1.

### Plasmid transfection and RNA interference

Plasmid transfections were performed with NEOFECT^TM^ DNA transfection reagent, and HighGene Transfection reagent (ABclonal, RM09014) was used for siRNA transfections. Specific custom siRNA was synthesized by GeneCreat. The siRNA sequence used for HDAC6 was 3ʹ-GGACAACATGGAGGAGGACAATGTA-5ʹ [14].

### CRISPR/Cas9 knockout

Three candidate gRNA-targeting LMNA exons were cloned into the lentiCRISPR-v2 vector. The target sequences were listed in Table S1. Each was co-transfected with packing plasmid pVSVg and psPAX2 into 293 T cells [15]. The suspension containing lentivirus was collected at 24, 48, and 72 h after transfection and concentrated by Ultrafiltration centrifuging. PLC/PFR/5 cells were incubated with the concentrated virus and polybrene (8 μg/mL). Puromycin was used to select positive cells for 48 h. The remaining cells were plated into a 96-well plate as single cells, then cultured and passaged into 24-well plates. These single cell colonies were determined by Western blotting and sequencing.

### In vivo assays

Female Balb/c nude mice were bought and fed in Tongji Medical College, Huazhong University of Science and Technology (Wuhan, China). A cell suspension containing 1 × 10^6^ cells of the indicated treatment was injected into the right axilla of the mice. The tumor size was measured and calculated as 0.5×length×width^2^. Tumors were obtained and weighed at the end of the experiments. Tumor tissues were fixed in 4% formaldehyde and handed to ServiceBio for HE or IHC staining. For in situ assays, the mice were anesthetized with isoflurane inhalation, and the liver lobes were exposed via a ~1-cm midline abdominal incision. Then, 2 × 10^5^ indicated cells suspended in 50 µL PBS were injected into the liver lobe. Liver lobes with tumors were obtained and weighed at the end of the experiments. The mice were intraperitoneally injected with NaCr at the dose of 12 mmol/kg body wt or 0.9% NaCl per 72 h.

### Statistical analysis

Quantified results are presented as the mean ± SEM of at least three duplicates. Comparisons between two groups were performed with an unpaired Student’s t-test. Results were considered statistically significant only if the P-value was less than 0.05, and the significant level is indicated as *P < 0.05, **P < 0.01, and ***P < 0.001.

Other methods are provided in the Supplementary Information.

## Results

### Lysine crotonylation associated with hypoxia contributes to liver cancer cell proliferation

**Fig. 1:**
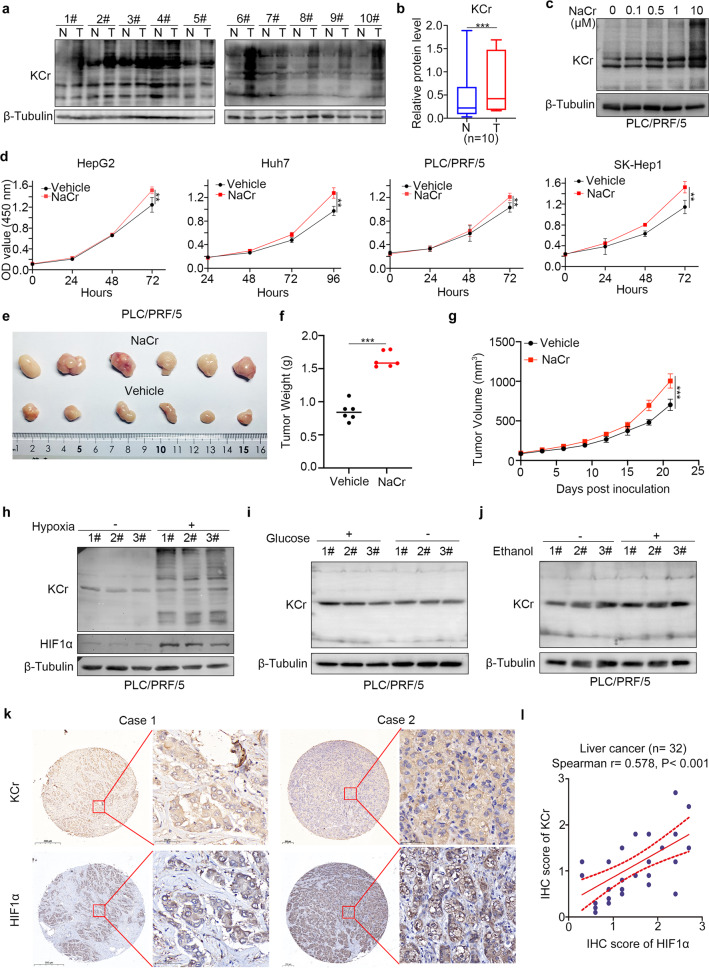
Lysine crotonylation associated with hypoxia contributes to liver cancer cell proliferation. **a**, **b** Kcr level of liver cancer tissue (T) and adjacent liver tissue (N) was measured by Western blot with pan-Kcr. Image J was applied to quantify the blots. Beta-tubulin served as an internal reference. ***,*P* < 0.001. **c** Western blot analysis of Kcr level in PLC/PRF/5 cells treated with NaCr at indicated concentration. **d** CCK-8 analysis of HepG2, Huh7, PLC/PFR/5, and SK-Hep1 cells treated with 10 mM NaCr or vehicle. Data presented as the mean ± SEM. *N* = 3, ***, *P* < 0.001. **e–g** Subcutaneous xenograft assay of PLC/PFR/5 cells treated with 12 mmol/kg body wt NaCr or vehicle (*N* = 6). Data presented as the mean ± SEM of three independent experiments. **, *P* < 0.01; ***, *P* < 0.001. **h**–**j** Western blot analyzed Kcr level of PLC/PRF/5 cells treated with indicated stimulus. Cells were cultured in 1% O_2_, cultured in glucose-free DMEM, or treated with 100 mM ethanol for 12 h. **k** HIF1α expression and Kcr level were detected with IHC in a tissue microarray. Typical image of IHC is shown in (**k**). **l** Spearman correlation analysis of IHC score was performed. *P* values are indicated.

Kcr was correlated with the tumor, node, and metastasis (TNM) stage of hepatocellular carcinoma and promoted the migration of liver cancer cells [16]. However, the specific role of Kcr in liver cancer is still unclear. We detected the Kcr level in 10 pairs of liver cancer tissues (T) and adjacent non-tumor liver tissues (N) from our hospital (Fig. 1a) and found that Kcr was relatively upregulated in the liver cancer tissues (Fig. 1b). In PLC/PRF/5 cells, treatment with a serial concentration of NaCr gradually increased the Kcr (Fig. 1c). We then showed that treating the liver cancer cell lines, HepG2, Huh7, PLC/PRF/5, and SK-Hep1 cells with 10 mM NaCr significantly promoted the cell growth in vitro (Fig. 1d). The subcutaneous xenograft assay revealed that NaCr enhanced the tumor growth in vivo (Fig. 1e, f, g). We subsequently explored why Kcr was involved in the progression of liver cancer. We detected the protein crotonylation of PLC/PRF/5 cells exposed to several potential cancer-promoting stimuli, including hypoxia, glucose starvation, and ethanol with pan-Kcr antibody. Occasionally, we found that the expression level of Kcr in PLC/PRF/5 was upregulated in hypoxia with three replicates (Fig. 1h, i, j). Intriguingly, the protein level of Kcr was positively correlated with HIF1α in the cohort of patients with liver cancer derived from a tissue microarray (patients with liver cancer *n* = 32, Spearman correlation *r* = 0.578, *P* < 0.001; Fig. 1k, l). This data suggested that Kcr correlated with HIF1α and promoted liver cancer cell proliferation.

### Identification of the proteins with Kcr in liver cancer

**Fig. 2:**
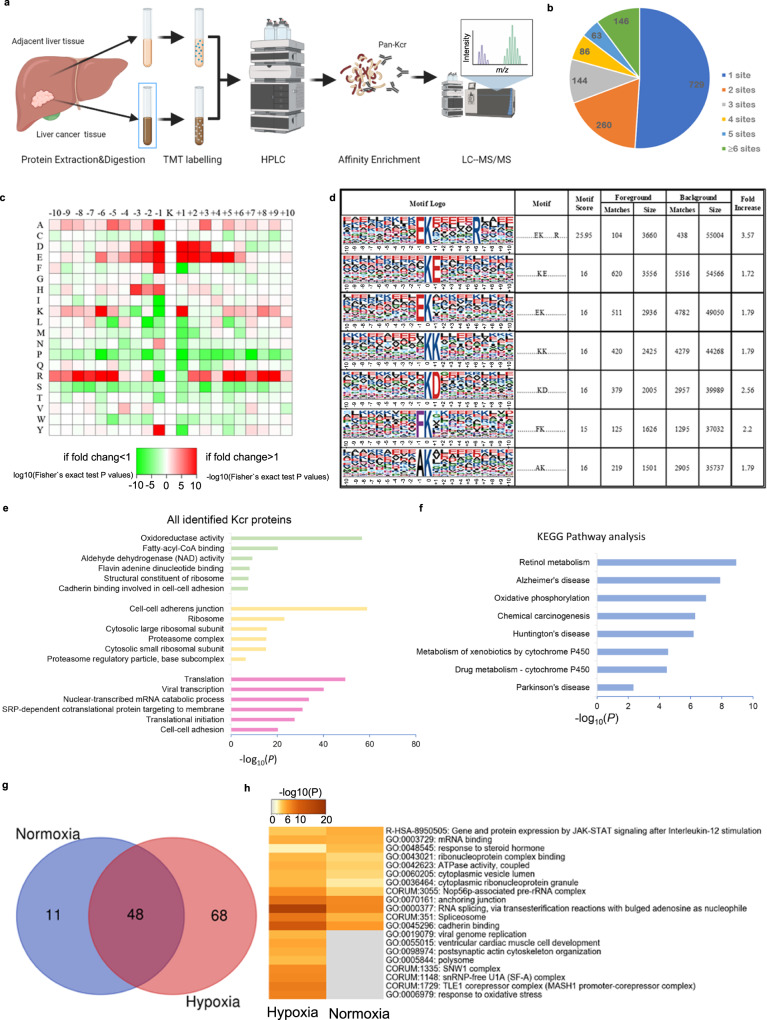
Identification of the proteins with Kcr in liver cancer. **a** A workflow schematic illustration showing TMT-labeled LC-MS/MS analysis of Kcr proteins in hepatocellular carcinoma and adjacent liver tissue. **b** The number of identified Kcr sites of each protein shown in pie chart. **c** Motif analysis of all indicated Kcr peptide. **d** Amino acid preference on both sides of the crotonylated lysine. **e**, **f** Gene Ontology and KEGG analysis of identified Kcr proteins. **g** The number of Kcr proteins identified in PLC/PRF/5 cells treated with hypoxia and normoxia. **h** Intracellular pathway enrichment analysis of the identified Kcr proteins in both groups.

To obtain a global view and quantitative analysis of liver cancer–related Kcr, TMT-labeled LC-MS/MS lysine crotonylome analysis was performed in 12 pairs of hepatocellular carcinoma and adjacent liver tissue (Fig. 2a). In total, 3,793 lysine crotonylation sites in 1,428 proteins were identified, of which 2,229 lysine crotonylation sites in 921 proteins were quantified. The proteins we identified were either single-site crotonylated or multi-site crotonylated. Around 729 (51.05%) proteins had a single Kcr site, and 146 (10.22%) proteins had more than six Kcr sites (Fig. 2b). We next analyzed the amino acids on both sides of the crotonylated lysine residue using iceLogo. We identified EKXXXXXR, XXXKEXXX, and XXXEKXXX as the significantly overrepresented hotspots for Kcr sites (Fig. 2c, d). Analysis of Kcr proteins by Gene Ontology (GO) and Kyoto Encyclopedia of Genes and Genomes (KEGG) pathway analysis revealed that Kcr proteins in liver and liver cancer tissue are widely involved in various cellular processes, including signaling, metabolism, translation, acylation, and carcinogenesis (Fig. 2e, f).

We quantified the changes of protein Kcr in liver cancer compared with adjacent liver tissue. The fold-change cutoff of quantitative ratios was set as above 1.5 or below 0.67. Our results reported that 222 sites in 114 proteins were upregulated and 94 sites in 68 proteins were downregulated in liver cancer tissue (Fig. S1a, b). GO analysis revealed that Kcr proteins in liver cancer were involved in ethanol oxidation, phagocytosis, cell adhesion, aging, and so on (Fig. S1c). KEGG pathway analysis indicated that upregulated proteins were highly enriched in the metabolism and chemical carcinogenesis process (Fig. S1d), which may be due to uprise of the special substrate crotonyl-CoA produced in the fatty acid metabolism process. Downregulated Kcr proteins were mainly related to infections (Fig. S1d). To further understand the crotonylation in response to hypoxia, we identified the Kcr proteins with IP-MS proteome analysis in pairs of hypoxic and normoxic PLC/PRF/5 cell lines (Fig. 2g). Fifty-nine proteins were identified in normoxic cells and 136 in hypoxic, with 48 co-identified (Fig. 2g). GO analysis found that Kcr proteins were actively involved in various cellular function processes, such as mRNA splicing, RNA metabolism, and response to oxidative stress (Fig. 2h). We then took an intersection of the Kcr proteins identified in liver cancer tissue quantitative analysis and hypoxic cell lines: CAT, S100A8, EEF2, and LMNA were co-identified (Fig. S1e).

### Lamin A is the key Kcr protein for regulating the proliferation of liver cancer cells

**Fig. 3:**
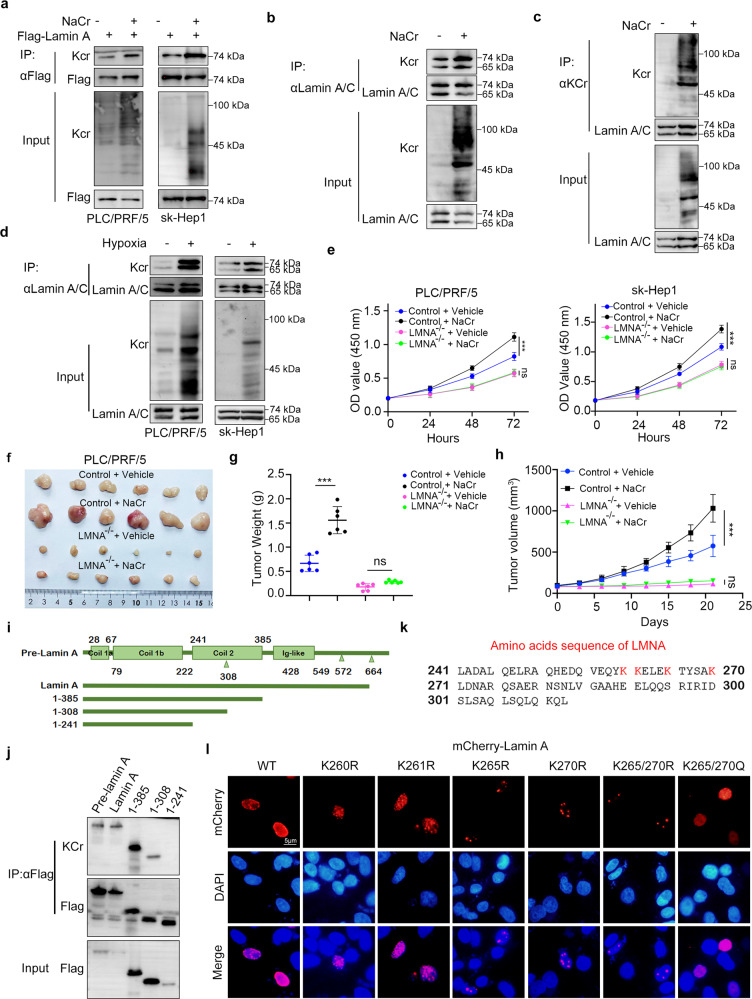
Characterization of crotonylation of lamin A. **a** Immunoprecipitation and Western blot analysis of crotonylation of lamin A with or without NaCr incubation. **b**, **c** Immunoprecipitation and Western blot analysis were performed to detect the crotonylation of endogenous lamin A. **d** Crotonylation of lamin A in hypoxia. **e** CCK-8 assays in PLC/PRF/5, sk-Hep1,PLC/PRF/5 LMNA^–/–^ and sk-Hep1 LMNA^–/–^ cells with or without NaCr incubation. Data presented as the mean ± SEM. *N* = 3, ns, not significant; ***, *P* < 0.001. **f** Subcutaneous xenograft assay of PLC/PRF/5 cells and PLC/PRF/5 LMNA^–/–^ cells treated with or without NaCr (*N* = 6). Data presented as the mean ± SEM. Ns, not significant; ***, *P* < 0.001. **g** Tumor weight of the subcutaneous xenograft at day 21 after inoculation. **h** Tumor volume of subcutaneous xenograft shown in line graph. **i** Schematic representation of lamin A motif and the recombinant of lamin A. **j** The Kcr of recombinant lamin A was analyzed by immunoprecipitation and Western blot. **k** 241–308 amino acids sequence of lamin A. **l** Immunofluorescence assay using anti-mCherry antibodies. NaCr was used at a concentration of 10 mM for in vitro assay and 12 mmol/kg body wt for in vivo assay.

Lamin A was an essential component of the nuclear lamina. Mutations or expression alterations of LMNA have been proven to be closely related to laminopathies and cancer. Lamin A has been documented to act as an oncogenic protein to enhance the proliferation of hepatocellular carcinoma [17]. We wondered whether lamin A was the key determinant for Kcr modification–induced tumor growth in liver cancer. We first found that 10 mM NaCr increased the Kcr modification of exogenously and endogenously expressed lamin A (Fig. 3a, b, c). We also noticed that hypoxia resulted in increasing the Kcr of lamin A (Fig. 3d).

We next engineered PLC/PRF/5 and sk-Hep1 cell lines with LMNA knockout using CRISPR/Cas9 to further explore the role of crotonylation of lamin A in hepatocellular cancer cells (Fig. S2a, b, c), and the LMNA-KO cells were marked as LMNA^-/-^. We then showed that knockout of LMNA could attenuate the tumor growth–promoting effect of NaCr treatment in cells and mice (Fig. 3e, f, g, h and Fig. S2d). This indicated that lamin A mediated the Kcr medication-induced proliferation of liver cancer cells. We also found that hypoxia slightly promotes cell growth in wild type liver cancer cells, however the affection disappeared in LMNA^–/–^ cells (Fig. S2e). We detected 17 potential crotonylation sites of lamin A in our LC-MS/MS data, but which sites were of function remained to be further confirmed. Moreover, we generated the recombinant of lamin A (Fig. 3i), and the recombinant containing 241–308 amino acids determined the Kcr medication of LMNA (Fig. 3j). The amino acid sequence of lamin A in the region of 241–308 included four potential crotonylated lysines (K260, K261, K265, and K270) (Fig. 3k). We substituted lysine (K) with arginine (R) to prevent Kcr at the corresponding specific sites in lamin A. Notably, the K265/270 R mutant of lamin A tremendously reduced the crotonylation of lamin A, which suggested that lamin A was crotonylated at the 265 and 270 sites.

### The crotonylation of lamin A promotes the proliferation of liver cancer cells

**Fig. 4:**
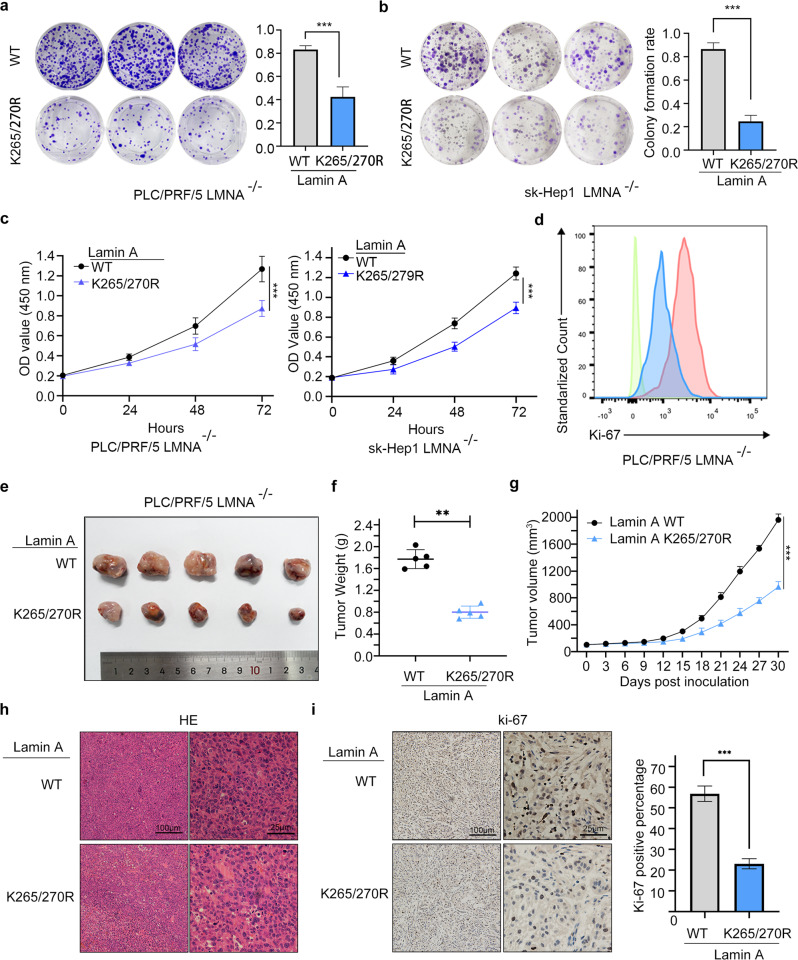
The crotonylation of lamin A promotes the proliferation of liver cancer cells in vivo and in vitro. Colony formation (**a**, **b**) and CCK-8 (**c**) assay in PLC/PRF/5 LMNA^–/–^ and sk-Hep1 LMNA^–/–^ cells with overexpression of lamin A WT or K265/270 R mutant. Data presented as the mean ± SEM. *N* = 3, ***, *P* < 0.001. **d** Ki-67 expression was analyzed by flow cytometry. Data presented as the mean ± SEM. *N* = 3, ***, *P* < 0.001. **e** Subcutaneous xenograft assay of PLC/PRF/5 LMNA^–/–^ cells with overexpression of lamin A WT or K265/270 R mutant (*N* = 5). **f** Tumor weight of the subcutaneous xenograft at day 30 after inoculation (*N* = 5). Data presented as the mean ± SEM. **, *P* < 0.01. **g** Tumor volume of subcutaneous xenograft shown in line graph (*N* = 5). Data presented as the mean ± SEM. ***, *P* < 0.001. **h** HE stain of the xenograft tumor tissue. **i** Ki-67 expression was detected by IHC. Data presented as the mean ± SEM. *N* = 5, ***, *P* < 0.001.

We overexpressed lamin A WT (wild-type) or Lamin A -K265/270 R mutants in LMNA^-/-^ cells. Colony formation assay, CCK-8 assay, and Ki-67 staining assay indicated that the K265/270 R mutant of lamin A with loss of the function of crotonylation decreased the capability of cell growth (Fig. 4a–d). Similarly, in vivo study demonstrated that the K265/270 R mutant of lamin A reduced the tumor growth ability of liver cancer cells (Fig. 4e–g). We also found that the K265/270 R mutant resulted in low Ki-67 staining levels in the xenograft tissues (Fig. 4h, i). These results showed that Kcr of lamin A enhanced liver cancer cell proliferation.

### HDAC6 decreased the crotonylation of lamin A

**Fig. 5:**
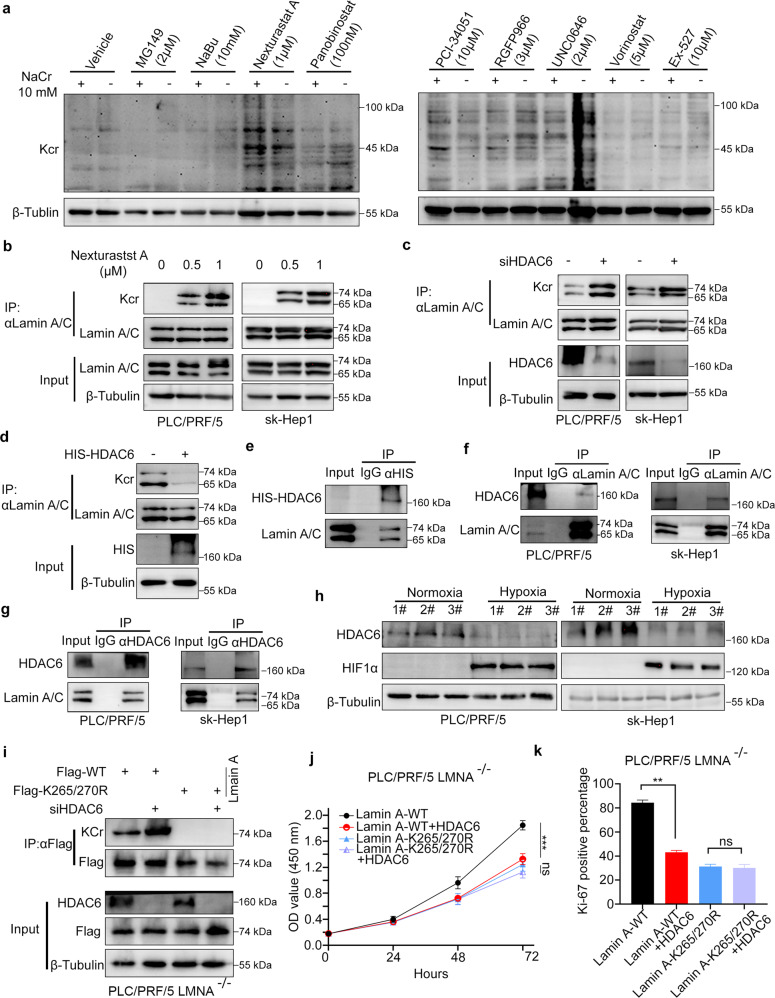
HDAC6 was associated with the crotonylation of Lamin A. **a** Kcr level of PLC/PRF/5 cells treated with small molecular inhibitors and NaCr (10 mM) was measured by Western blot. The crotonylation of lamin A with indicated concentration of Nexturastat A (**b**), knock down of HDAC6 (**c**) or overexpression HDAC6 (**d**) was detected by IP followed with Western blot. **e**–**g** The exogenous and endogenous interaction of HDAC6 and lamin A was determined by co-immunoprecipitation (Co-IP). **h** HDAC6 expression in hypoxia and normoxia was detected by Western blot. **i** The crotonylation of exogenous WT or mutant lamin A in LMNA-KO PLC/PRF/5 cells with or without knock-down of HDAC6. CCK-8 assay (**j**) AND Ki-67 expression (**k**) in LMNA-KO PLC/PRF/5 cells with overexpression of WT of mutant lamin A and HDAC6. Data presented as the mean ± SEM. *N* = 3, ns, not significant; **, *P* < 0.01; ***, *P* < 0.001.

A previous study reported that HDAC silencing or adding HDAC inhibitors could increase the crotonylation of liver cancer cells [16]. Here, we treated the PLC/PRF/5 cells with distinct HDAC inhibitors or histone acetyltransferase inhibitors as indicated (Fig. 5a). We demonstrated that a specific HDAC6 inhibitor, Nexturastat A, increased the crotonylation of liver cancer cells after NaCr treatment (Fig. 5a). Importantly, Nexturastat A increased the crotonylation of lamin A in a dose-dependent manner in PLC/PRF/5 and sk-Hep1 cells (Fig. 5b). Unsurprisingly, knockdown of HDAC6 elevated the crotonylation of lamin A (Fig. 5c), but overexpression of HDAC6 reduced the crotonylation of lamin A (Fig. 5d). Moreover, we showed that ectopically expressed HDAC6 bound with lamin A in 293 T cells (Fig. 5e). Lamin A and HDAC6 reciprocally interacted in PLC/PRF/5 and sk-Hep1 cells (Fig. 5f, g). Interestingly, we found that the low oxygen condition could lead to the downregulation of HDAC6 in PLC/PRF/5 and sk-Hep1 cells with three replicates (Fig. 5h). Furthermore, we showed that HDAC6 silencing specifically enhanced the Kcr of lamin A at the 265 and 270 sites (Fig. 5i). Finally, we found that overexpression of HDAC6 could diminish the growth-promoting effect of lamin A induced by the Kcr modification in PLC/PRF/5 LMNA^-/-^ cells (Fig. 5j, k).

### HDAC6/lamin A complex regulated the senescence of liver cancer cells

**Fig. 6:**
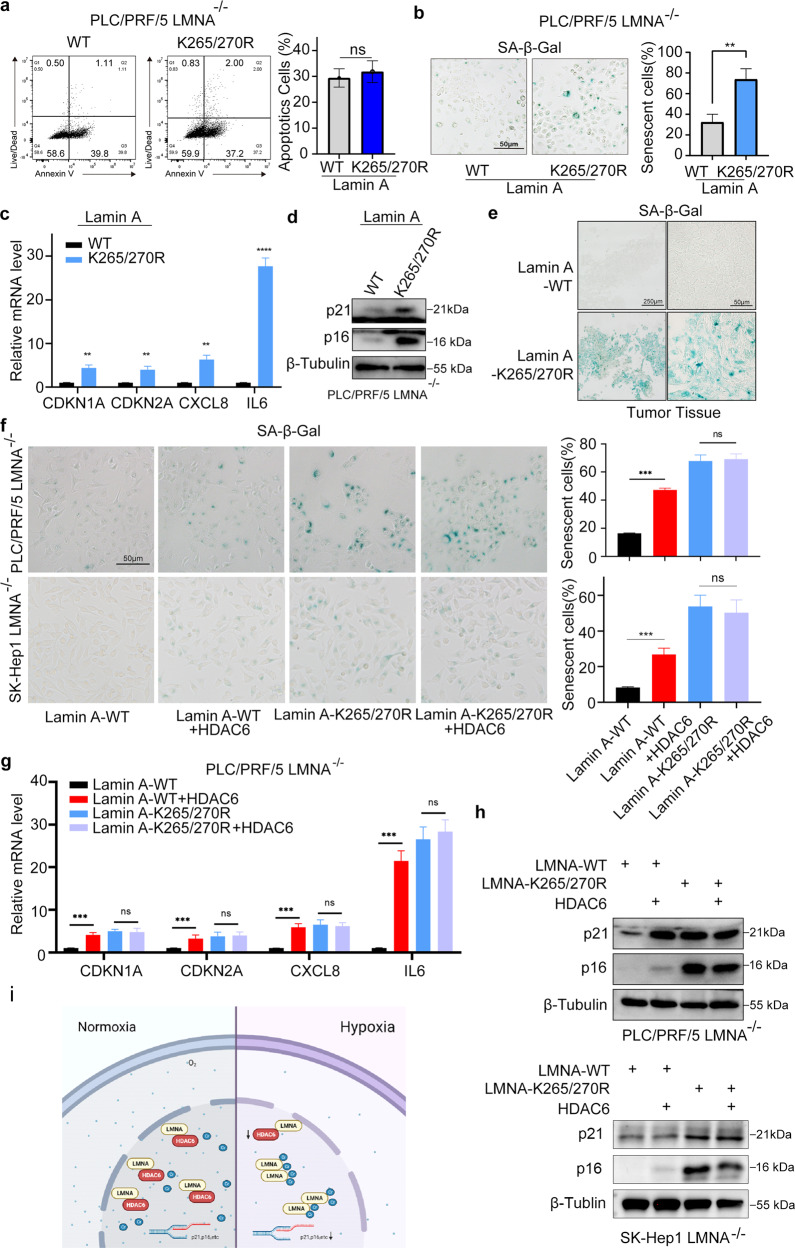
HDAC6/lamin A complex regulated the senescence of liver cancer cells. **a** PE/annexin-V staining of PLC/PRF/5 LMNA^–/–^ cells with the overexpression of WT or mutant lamin A. Data presented as the mean ± SEM. *N* = 3, ns, not significant. **b** SA-β-Gal staining of PLC/PRF/5 LMNA^–/–^ cells with the overexpression of WT or mutant lamin A. Data presented as the mean ± SEM. *N* = 3, **, *P* < 0.01. **c** Relative mRNA levels of p21, p16, IL-6, and IL-8 were quantified with qPCR. Data presented as the mean ± SEM. *N* = 3, **, *P* < 0.01; ***, *P* < 0.001. **d** The expression of p21 and p16 was detected by Western blot. **e** SA-β-Gal staining of subcutaneous xenograft tumor tissue. **f** SA-β-Gal staining of sk-Hep1 LMNA^–/–^ and PLC/PRF/5 LMNA^–/–^ cells with overexpression of WT or mutant lamin A and HDAC6. Data presented as the mean ± SEM. *N* = 3, ns, not significant; ***, *P* < 0.001. **g** The relative mRNA levels of p21, p16, IL-6, and IL-8 were quantified by qPCR. Data presented as the mean ± SEM. *N* = 3, ns, not significant; ***, *P* < 0.001. **h** The expression of p21 and p16 was detected with Western blot. **i** A hypothetical model showing that lamin A crotonylation was upregulated in hypoxia due to the downregulation of HDAC, leading to liver cancer senescence bypassing and proliferation.

The question was raised of how the Kcr of the lamin A complex regulated the proliferation of liver cancer cells. The apoptotic assay with PE/annexin-V staining showed that the K265/270 R mutant of lamin A did not change the apoptotic rate of liver cancer cells compared to lamin A WT (Fig. 6a). Recent studies have clarified the irreplaceable role of crotonylation in cellular senescence. We next checked whether Kcr of lamin A affected the cellular senescence. Notably, we showed that the lamin A K265/270 R mutant increased the cellular senescence marker (SA-β-Gal) and corresponding senescence-related genes, including p21, p16, IL-6, and IL-8 (Fig. 6b–d). This phenomenon was also observed in the xenograft tissues (Fig. 6e). We also noticed that hypoxia promotes liver cancer cell growth and restrains cellular senescence harboring wild type lamin A but not lamin A-K265/270 R. Moreover, we showed that overexpression of HDAC6 increased the senescence in lamin A WT expression cells, but co-overexpression of the lamin A K265/270 R mutant and HDAC6 could not further increase the senescence cells compared with lamin A K265/270 R mutant expression alone (Fig. 6f–h). Together, these data suggested that the HDAC6/ lamin A complex modulated the senescence of liver cancer cells.

## Discussion

In the current study, we demonstrated that protein crotonylation positively responded to hypoxia in liver cancer and contributed to liver cancer cell proliferation. We characterized global crotonylome in hepatocarcinoma by using quantitative proteomics. We identified 3,793 lysine crotonylation sites in 1,428 proteins and several overrepresented hotspots for Kcr sites. We further used IP-MS to identify Kcr proteins in response to hypoxia. Taking an intersection of these data, we found lamin A as a co-identified protein. We reported K265 and K270 as the crotonylation sites in lamin A. K265/270 crotonylation plays a vital role in lamin A function regulation by maintaining its appropriate subcellular positioning. We then found that the K265/270cr promoted the proliferation of liver cancer by inducing senescence. Inhibitor screening suggested HDAC6 as a decrotonylase of lamin A. We showed that the interaction of HDAC6 and lamin A contributed to its crotonylation. HDAC6 was downregulated in hypoxia and thus led to an increase in lamin A crotonylation and liver cancer proliferation.

Crotonylation is a newly discovered acetylation of which the short-chain fatty acids and co-enzyme are substrates. Earlier studies reported that fatty acid metabolism was closely involved in crotonylation regulation. Fellows et al. found that short-chain fatty acids derived such as butyrate in the intestine promote histone crotonylation by inhibiting HDACs and providing substrates such as crotonyl-CoA [18]. Histone crotonylation is also highly sensitive to fatty-acid oxidation pathway status [19]. Histone crotonylation is downregulated in the mPFC of susceptible rodents exposed to CSDS due to the upregulation of decrotonylase CDYL, according to Liu et al. [20]. In our study, we demonstrated that protein crotonylation is highly sensitive to oxygen concentration fluctuation. Hypoxia, rather than sugar starvation or ethanol exposure, promotes crotonylation in PLC/PRF/5 cells. Protein crotonylation level positively correlates to HIF1α expression in liver cancer. Our IP-MS also showed that more proteins are crotonylated in hypoxic conditions. Further work remains to determine whether hypoxia regulates crotonylation by influencing the short fatty acid metabolism flux and whether this regulation is universally applicable.

Previous studies on non-histone crotonylation with the application of quantitative proteomics have usually been carried out on cell lines [12, 21]. This gives better data stability and uniformity. In the current study, we performed quantitative lysine crotonylome analysis on 12 pairs of hepatocellular carcinoma and adjacent liver tissue. We identified 3,793 lysine crotonylation sites in 1,428 proteins and showed that Kcr was upregulated in liver cancer compared with adjacent liver tissue. Our data suggested that EKXXXXXR, XXXKEXXX, and XXXEKXXX occurred most frequently in Kcr peptides, which was highly consistent with previous reports [7]. This is the only study to our knowledge to profile liver cancer crotonylation with quantitative proteomics; it showed good congruence with recent studies. To further investigate the relevance of crotonylation involved in hypoxia and liver cancer, we took an intersection of Kcr proteins identified in liver cancer tissue and hypoxic cell lines and came to several proteins, among which lamin A was most related to liver cancer senescence and proliferation [17]. Whether the crotonylation of CAT, S100A8, and EEF2 plays a vital role in liver cancer proliferation and the cellular response to hypoxia remains to be investigated.

Lamin A, encoded by the LMNA gene and processed by ZMPSTE24 for maturation, is an essential component of the nuclear envelope [22]. The mutation of LMNA can lead to pathological states including dilated cardiomyopathy, Hutchinson-Gilford progeria syndrome (HGPS), and atypical progeria syndrome [23]. Most of these mutations result in the maturation dysfunction and accumulation of prelamin A at the nuclear envelope [24], eventually causing impaired DNA damage repair, cellular senescence, and dysfunction [25–27]. The sublocation of lamin A is important to maintain its normal function [28, 29]. Mottioli et al. reported that impaired lamin A/C interactions with HDAC2 during oxidative stress in HGPS resulted in p21 expression and induced cellular and organism aging [30]. Our study showed that the crotonylation of lamin A occurred at K265 and K270, and the prevention of crotonylation of lamin A by mutating K to R at these sites results in the disposition of lamin A. K265/270 R mutants did not show maturation abnormality because the proteins formed speckles rather than gathering at the nuclear envelope. However, preventing the crotonylation of lamin A at K265/270 led to cellular senescence and cell proliferation suppression by inducing p21 and p16 expression. Future work should include the verification of potential regulatory targets of lamin A crotonylation and pathways leading to p21 and p16 expression.

Classic histone acetyltransferases have crotonyltransferase, whereas histone deacetylases relate to decrotonylation activity, especially class I HDACs and SIRTs [11, 31]. Yu et al. found that CDYL was a typical crotonyl-CoA hydratase to negatively regulate histone Kcr [7]. In this study, we reported a novel decrotonylase HDAC6 and characterized its interaction with lamin A. HDAC6 decrotonylated lamin A at 265/270 lysine, maintaining its normal position and function. This is disrupted in hypoxia, where HDAC6 is downregulated and leads to lamin A crotonylation and promotion of liver cancer proliferation. For future study, we must investigate the potential crotonylases of lamin A, which will help us understand the function and regulation of lamin A crotonylation.

In summary, we demonstrate that protein crotonylation is upregulated in hypoxia and plays a vital role in liver cancer progression. We also identified lamin A as a crotonylated protein at K265/270, regulated by HDAC6. Lamin A K265/270cr plays a vital role in liver cancer senescence and proliferation regulation. Controversy remains as to whether lamin A is the only crotonylated protein regulating cancer cell growth in response to hypoxia. Studying the crotonylases working in hypoxia will also be interesting. Further research on the function and regulation of non-histone crotonylation is eagerly anticipated.

## Supplementary information

Supplementary information

Original Data File

aj-checklist

## Data Availability

The datasets used and/or analyzed during the current study are available from the corresponding authors on reasonable request.
