# Supplementary material for: Global crotonylome reveals hypoxia-mediated lamin A crotonylation regulated by HDAC6 in liver cancer
Source: Cell Death Dis. 2022 Aug 17;13(8):717. doi: 10.1038/s41419-022-05165-1 (PMC9385620; doi:10.1038/s41419-022-05165-1)
Supplement: Supplementary file 1 — Supplementary information [file 41419_2022_5165_MOESM1_ESM.docx]

**Global crotonylome reveals hypoxia–mediated LMNA crotonylation regulated by HDAC6 in liver cancer**

**Supplementary fig. 1. Crotonylome analysis of Kcr proteins in liver cancer**

a. Ratio distribution of quantified Kcr sites between liver cancer tissue and adjacent liver tissue shown in histogram. b. The quantification of Kcr sites in relation to peptide intensities shown in scatterplot. c. GO analysis of all quantified Kcr proteins potentially associated with liver cancer shown in bar graph. d. KEGG pathway associated with up-regulated and down-regulated Kcr proteins in liver cancer shown in bar graph. e. Veen diagram showing the Kcr proteins up- or down-regulated in liver cancer tissue and hypoxia. f. Proteins co-identified in two independent LC-MS/MS analysis.


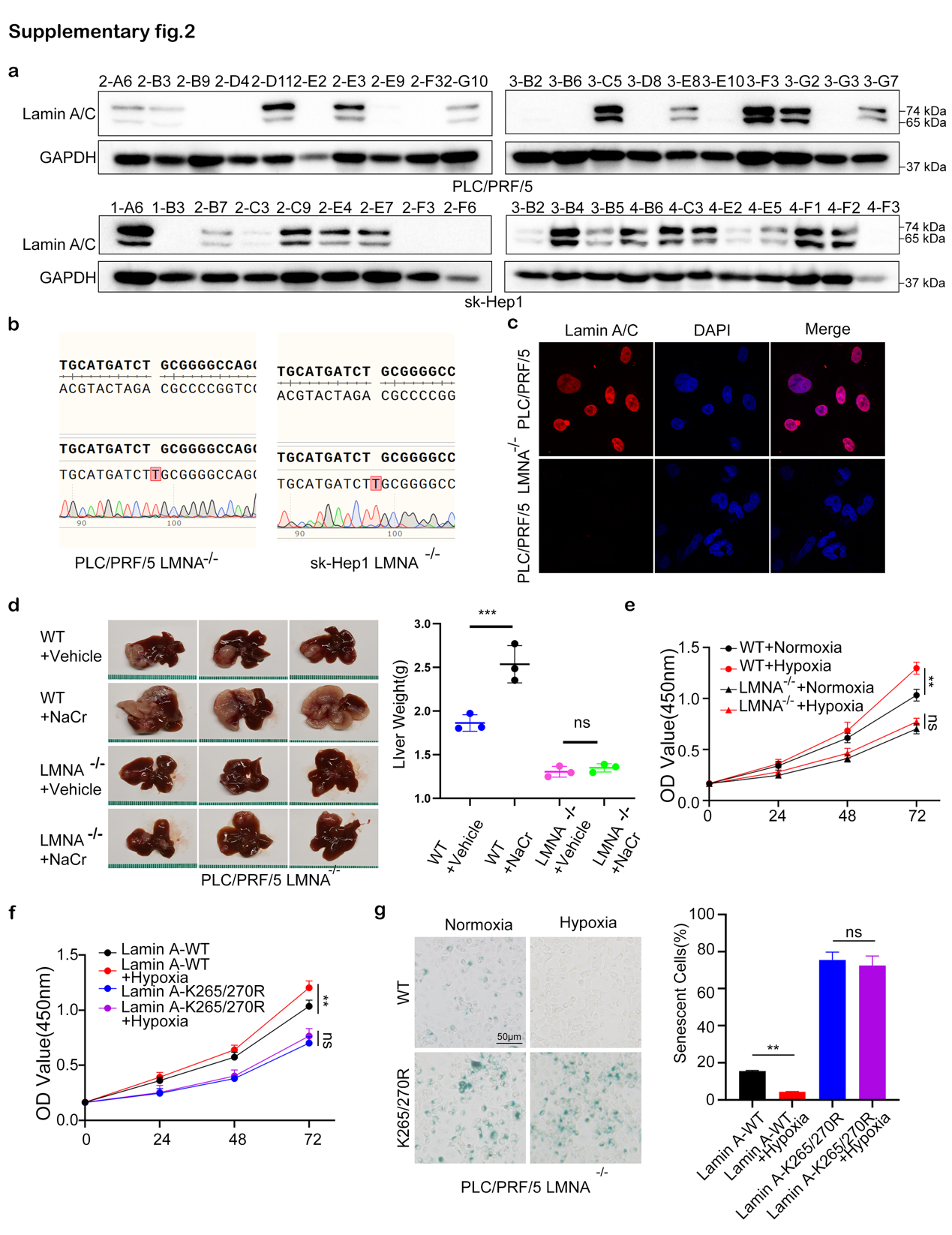


**Supplementary fig. 2. Construction of LMNA^-/-^ cell lines and in situ assays.**

a. Western Blot analysis detecting expression of LMNA in monoclonal cell lines stable transfected with LentiCRISPRv2. b. Nucleic acid sequencing of LMNA exon5. c. LMNA expression of monoclonal cell lines by Immunofluorescence detection. d. Orthotopic allograft assay of WT and LMNA^-/-^ cells treated with or without NaCr(N=3). NaCr was given at the dose of 12 mmol/kg body wt every 3 days. e. CCK-8 assays in PLC/PRF/5 and PLC/PRF/5 LMNA^-/-^ cells treated with hypoxia or normoxia. f. CCK-8 assays in PLC/PRF/5 LMNA^-/-^ cells harboring lamin A and lamin A-K265/270R treated with hypoxia or normoxia. g. SA-β-Gal staining of PLC/PRF/5 LMNA^-/-^ cells with overexpression of WT and mutant lamin A treated with hypoxia or normoxia.


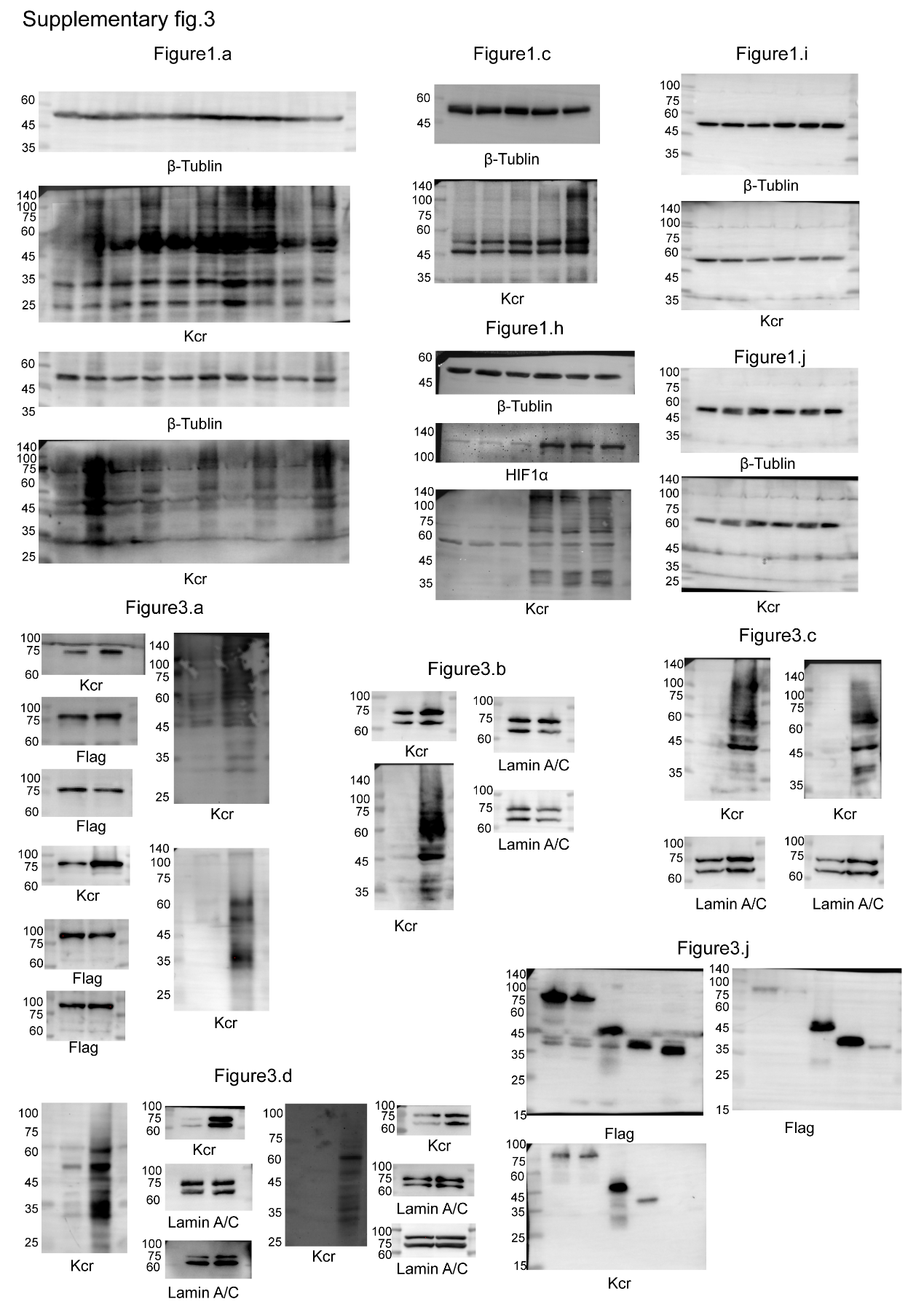


**Supplementary fig. 3. Full scans of western blots of figure1 and figure3.**

**
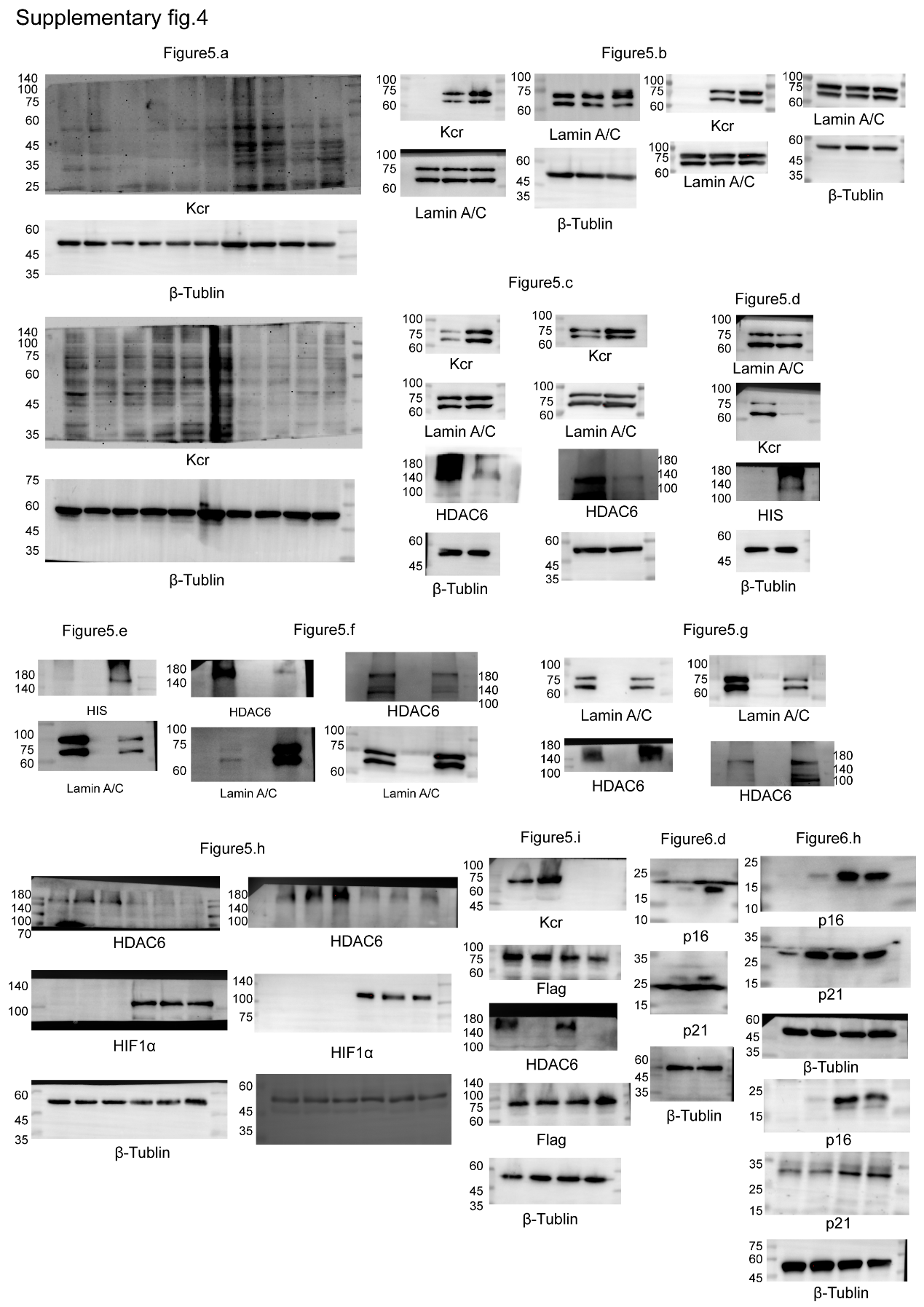
**

**Supplementary fig. 4. Full scans of western blots of figure5 and figure6.**


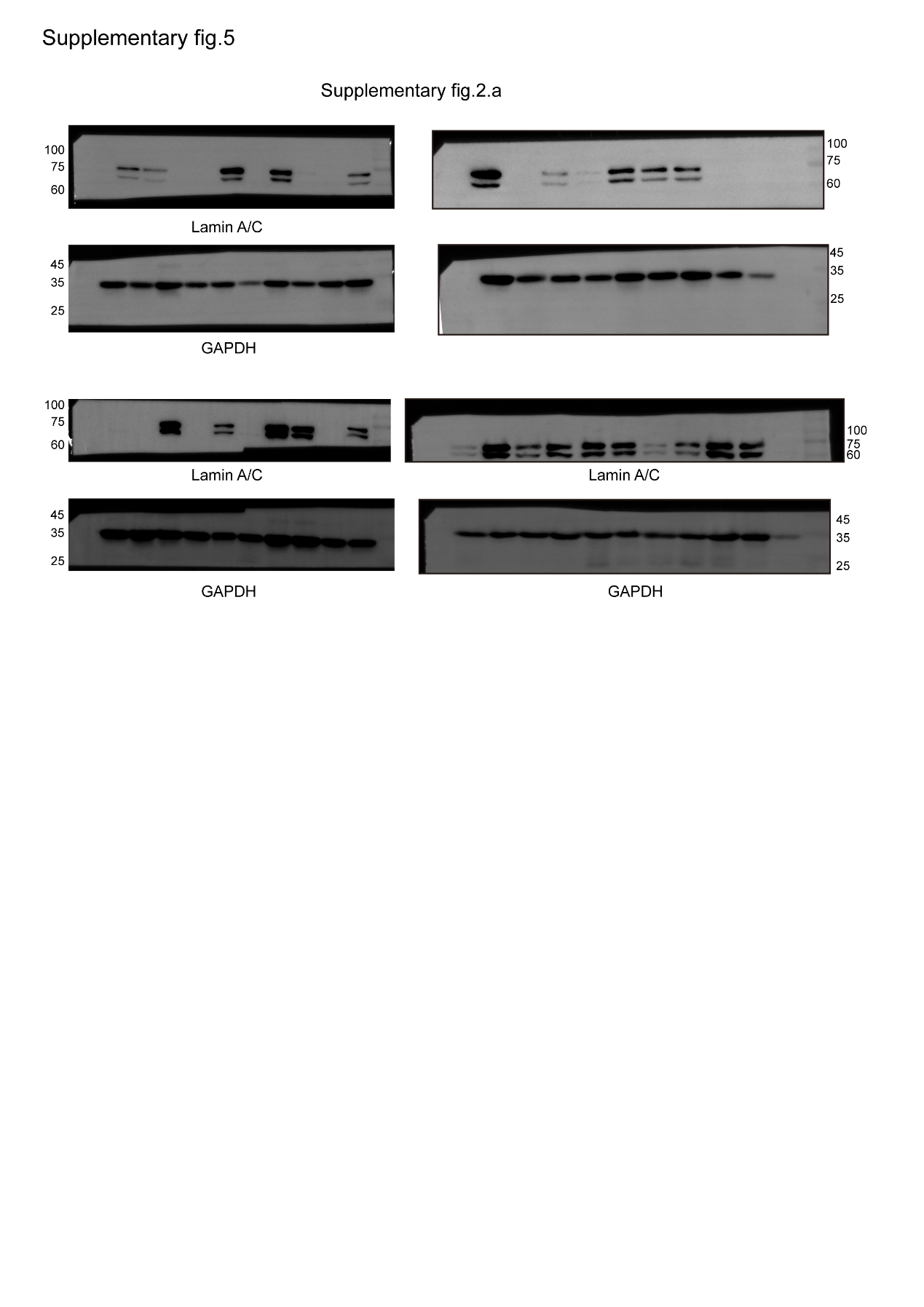


**Supplementary fig. 5. Full scans of western blots of Supplementary fig.2.**

**Table. S1 The sequence of primers used in this study.**

|  | Sequence |
| --- | --- |
| LMNA taregt 1 | 5'-TCTCAGTGAGAAGCGCACG-3' |
| LMNA taregt 2 | 5'-GGCGAGCTGCATGATCTGC-3' |
| LMNA taregt 3 | 5'-GGCTCTGCTGAACTCCAAGG-3' |
| LMNA sequencing primer | 5'-AGGAGGGTGACCTGATAGC-3' |
|  |  |
| CDKN1A-F | 5'-CGATGGAACTTCGACTTTGTCA-3' |
| CDKN1A -R | 5'-GCACAAGGGTACAAGACAGTG-3' |
| CDKN2A -F | 5'-GGGTTTTCGTGGTTCACATCC-3' |
| CDKN2A -R | 5'-CTAGACGCTGGCTCCTCAGTA-3' |
| IL6-F | 5'-ACTCACCTCTTCAGAACGAATTG-3' |
| IL6-R | 5'-CCATCTTTGGAAGGTTCAGGTTG-3' |
| CXCL8-F | 5'-TTTTGCCAAGGAGTGCTAAAGA-3' |
| CXCL8-R | 5'-AACCCTCTGCACCCAGTTTTC-3' |
|  |  |
| Flag-LMNA-F | 5'-GACTGGATCCATGGAGACCCCGTCCCAG-3' |
| Flag-LMNA-R | 5'-GACTCTCGAGTCAGTAGGAGCGGGTGACCAGA-3' |
| Flag-preLMNA-R | 5'-GACTCTCGAGTTACATGATGCTGCAGTTCTGG-3' |
| Flag-LMNA1-385aa-R | 5'-GACTCTCGAGTTACTCCTCCTCGCCCTCCAA-3' |
| Flag-LMNA1-308aa-R | 5'-GACTCTCGAGTTACTGGCTGAGCTGGGCAGA-3' |
| Flag-LMNA1-241aa-R | 5'-GACTCTCGAGTTACAGCCGGCTCTCAAACTCAC-3' |
| mCherry-LMNA-F | 5'-TGCACTCGAGTCATGGAGACCCCGTCCCAG-3' |
| mCherry-LMNA-R | 5'-GATCGGATCCTTACATGATGCTGCAGTTCTGG-3' |
| Mut-LMNA K260R-F | 5'-TGGAGCAGTATAGGAAGGAGCTGGAG-3' |
| Mut-LMNA K260R-R | 5'-CTCCAGCTCCTTCCTATACTGCTCCA-3' |
| Mut-LMNA K261R-F | 5'-TGGAGCAGTATAAGAGGGAGCTGGAG-3' |
| Mut-LMNA K261R-R | 5'-CTCCAGCTCCCTCTTATACTGCTCCA-3' |
| Mut-LMNA K265R-F | 5'-AGGAGCTGGAGAGGACTTATTCTG-3' |
| Mut-LMNA K265R-R | 5'-CAGAATAAGTCCTCTCCAGCTCCT-3' |
| Mut-LMNA K270R-F | 5'-TTCTGCCAGGCTGGACAATGCC-3' |
| Mut-LMNA K270R-R | 5'-GGCATTGTCCAGCCTGGCAGAA-3' |
| Mut-LMNA K265/270R-F | 5'-GGAGAAGACTTATTCTGCCAAGCTGGACA-3' |
| Mut-LMNA K165/270R-R | 5'-GGCATTGTCCAGCCTGGCAGAATAAGTCCTCTCCAGCTC-3' |
| Mut-LMNA K265/270Q-F | 5'-GAGCTGGAGCAGACTTATTCTGCCCAGCTGGACAATGCC-3' |
| Mut-LMNA K165/270Q-R | 5'-GGCATTGTCCAGCTGGGCAGAATAAGTCTGCTCCAGCTC-3' |
| 6*HIS-HDAC6-F | 5'-CACCACGGAGGAGGAAAGCTTATGAGTGGAGCGAACCG-3' |
| 6*HIS-HDAC6-R | 5'-TAAACGGGCCCTCTAGACTCGAGTTAGTGTGGGTGGGGCATATCCTCCCCA-3' |

The sequence details of the primers used in qPCR and plasmid construction.
