# Supplementary material for: Global crotonylome reveals hypoxia-mediated lamin A crotonylation regulated by HDAC6 in liver cancer
Source: Cell Death Dis. 2022 Aug 17;13(8):717. doi: 10.1038/s41419-022-05165-1 (PMC9385620; doi:10.1038/s41419-022-05165-1)

Supplementary fig.3

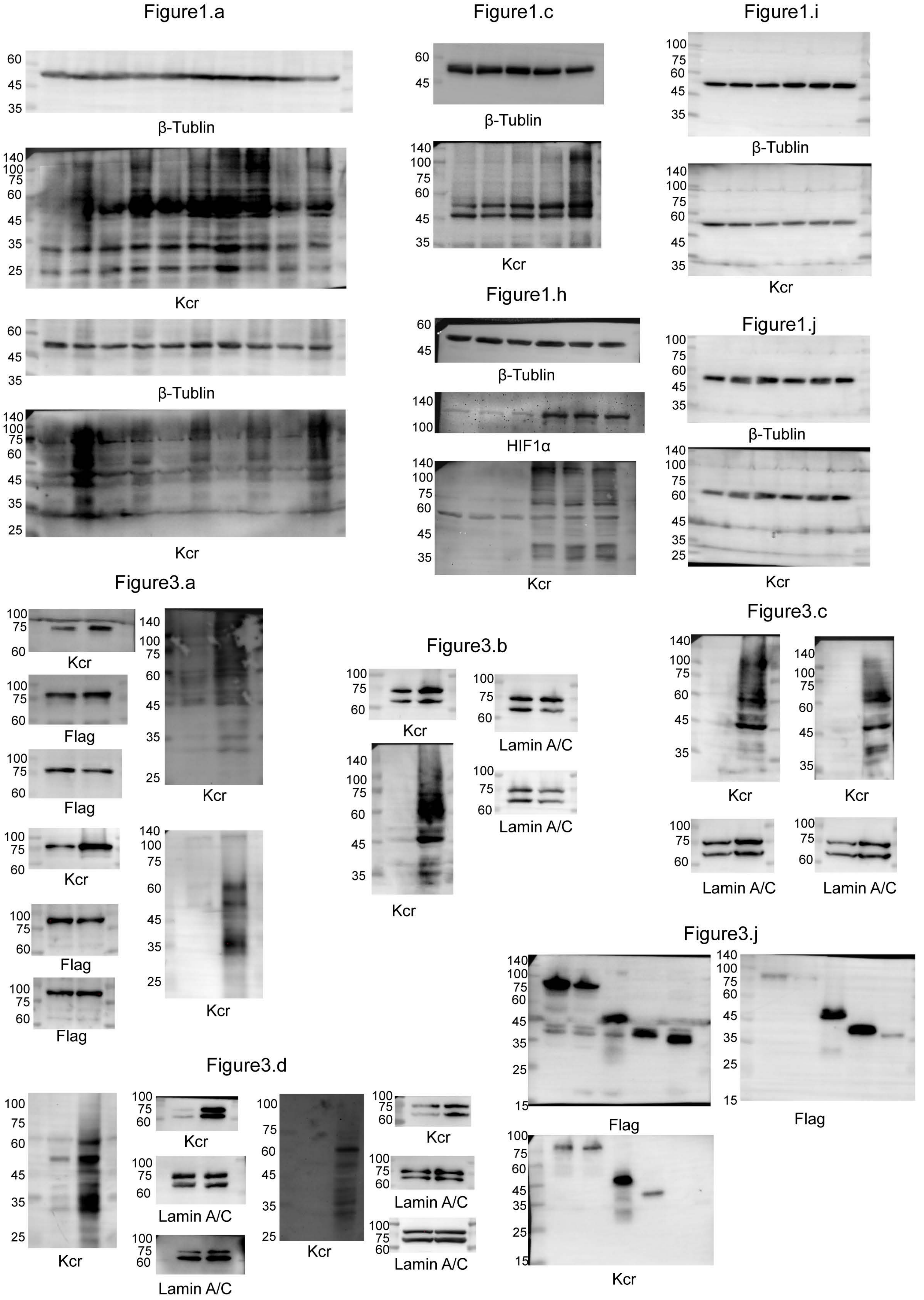

Supplementary fig.4

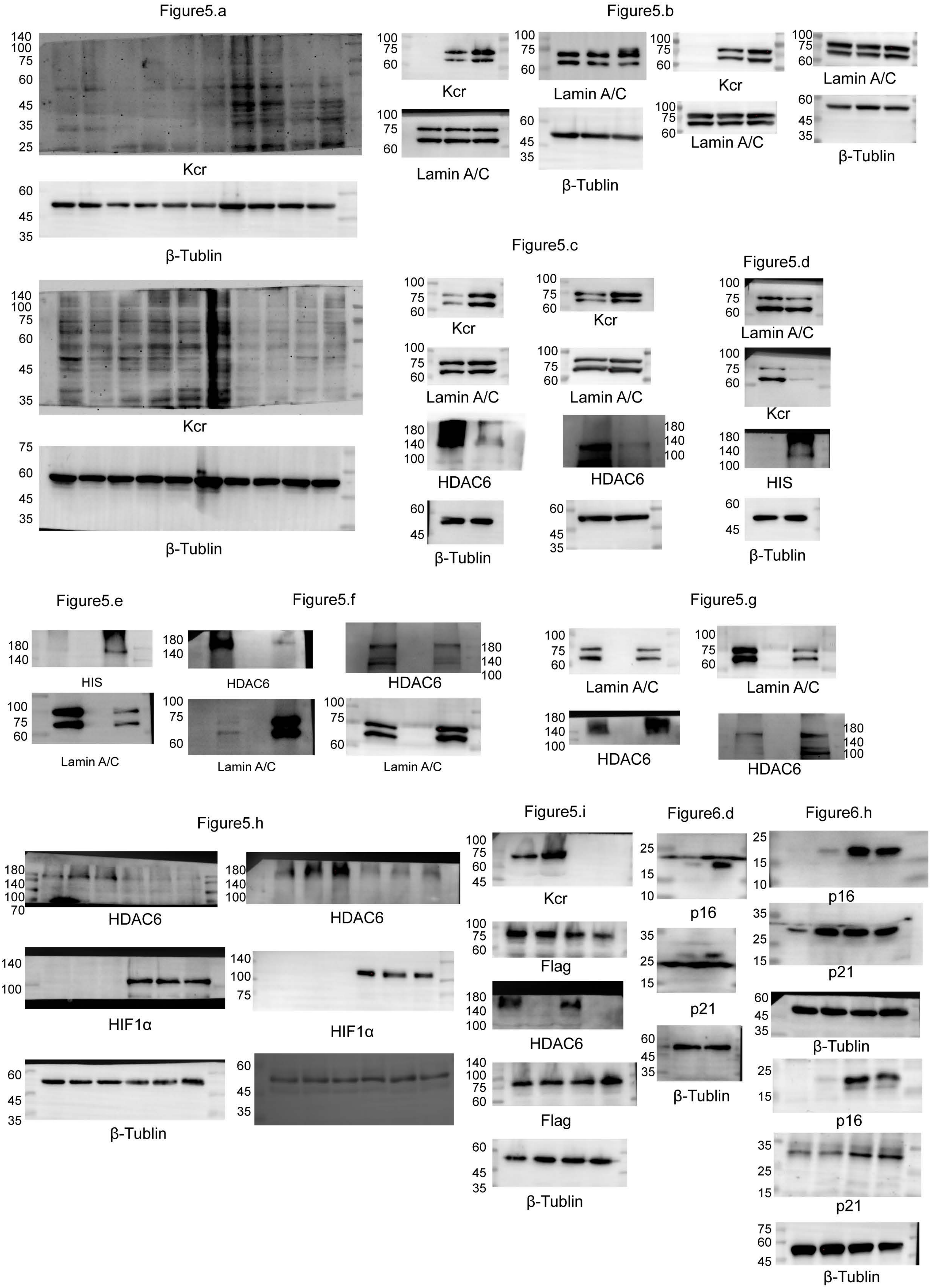

Supplementary fig.5

Supplementary fig.2.a

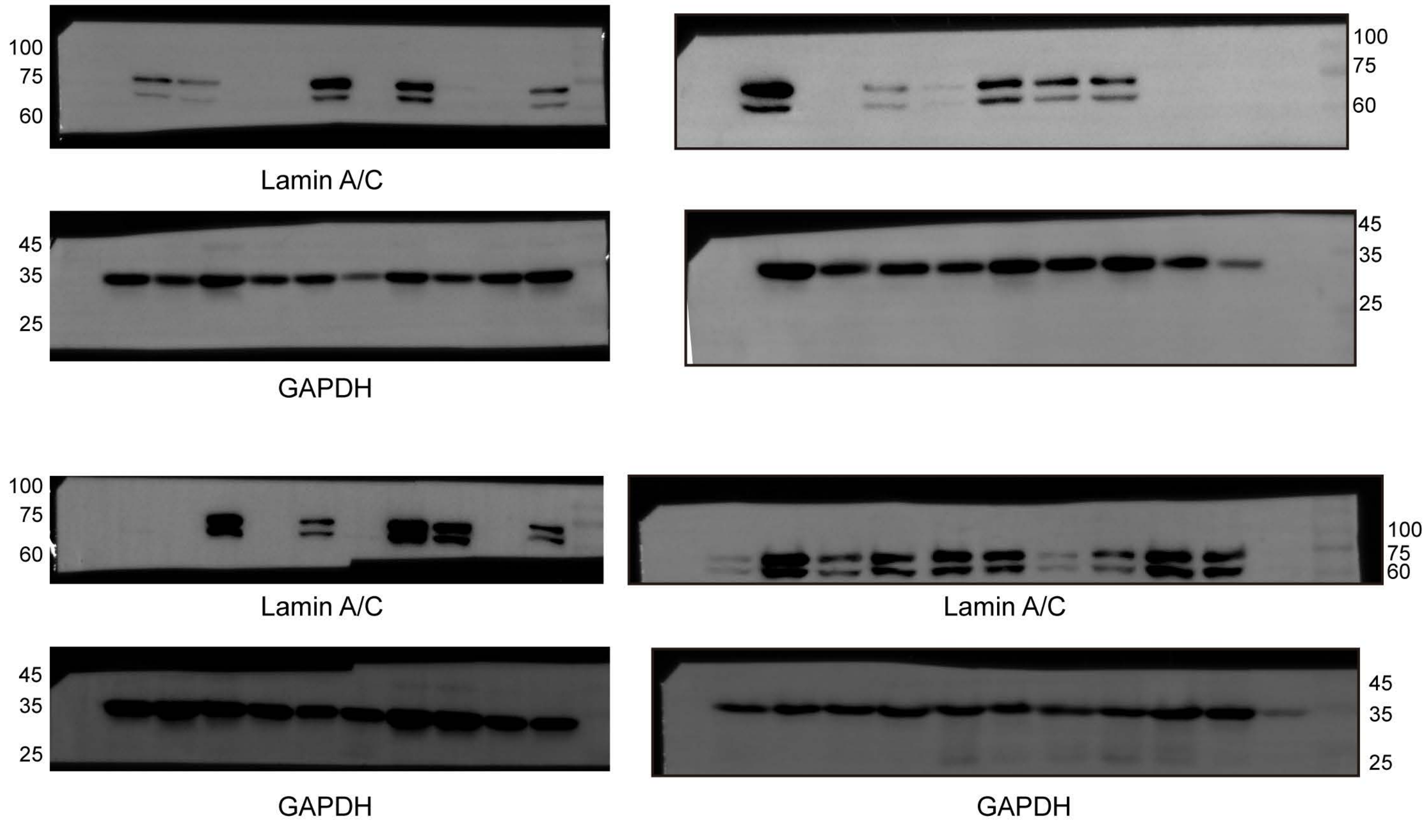

Supplement: Supplementary file 2 — Original Data File [file 41419_2022_5165_MOESM2_ESM.pdf]
